# Clinical and quality of life improvement after bilastine treatment among patients with autoimmune chronic spontaneous urticaria

**DOI:** 10.1371/journal.pone.0326445

**Published:** 2025-08-25

**Authors:** My Tra Thi Nguyen, My Huyen Le, Minh Nguyet Vu, Katrine Baumann, Per Stahl Skov, Doanh Huu Le

**Affiliations:** 1 Hue University of Medicine and Pharmacy, Hue University, Vietnam; 2 Hanoi Medical University, Hanoi, Vietnam; 3 National Dermatology and Venereology Hospital, Hanoi, Vietnam; 4 RefLab ApS, Hvidovre, Denmark; University of Split Faculty of Medicine: Sveuciliste u Splitu Medicinski fakultet, CROATIA

## Abstract

**Background:**

This study aimed to evaluate the clinical effectiveness of bilastine in type IIb autoimmune CSU (type IIb aiCSU) patients over an 8-week period, as well as to identify factors predicting treatment response.

**Method:**

34 type IIb aiCSU patients with positive basophil histamine release assays (BHRA) and positive Autologous Serum Skin Test (ASST) tests, from the Vietnam National Dermatology and Venereology Hospital, were included. Patients began treatment with the standard dose of bilastine, with the dose increased every two weeks for those who did not achieve adequate response. The Urticaria Control Test (UCT), Weekly Urticaria Activity Score (UAS7), and the Chronic Urticaria Quality of Life Questionnaire (CU-Q2oL) were used.

**Results:**

Significant improvements in all measures were observed, with 82.4% of patients achieving complete response by week 8. Of these, 47.1% responded to the standard dose, while 14.7% did not respond even at the maximum (x4) dose. UAS7 average scores decreased from a baseline mean of 28.9 to 2.9, indicating substantial reduction in disease activity. UCT scores improved from 47.1% of patients having poor disease control (UCT < 12) after week 2 to 85.3% achieving good control (UCT ≥ 12) by week 8. QoL also significantly improved, with CU-Q2oL scores dropping from 44.9 at baseline to 25.3 at week 8. Basopenia, high baseline UAS7 score and a history of autoimmune disease were associated with poorer treatment responses, while normal/high IgE levels were linked to better outcomes.

**Conclusion:**

The findings suggest that bilastine is highly effective in controlling aiCSU symptoms and improving QoL.

## Introduction

Chronic spontaneous urticaria (CSU) is a common dermatological condition defined by the recurrent appearance of wheals, angioedema, or both, lasting more than six weeks without identifiable external triggers, affecting 0.5% to 1% of the global population [1]. The condition’s symptoms, including severe itching and swelling, significantly impair daily functioning, sleep, and social activities, leading to a profound psychosocial and economic burden [2–4]. A distinct subgroup of CSU, known as type IIb autoimmune CSU (aiCSU), is defined by the presence of functional autoantibodies, such as IgG (IgA, IgM) anti-IgE or IgG anti-FcεRIα, which activate mast cells through immunologic mechanisms. These autoantibodies stimulate degranulation of mast cells independently of allergen exposure, leading to the release of histamine and other inflammatory mediators [5–7]. Diagnostic markers for aiCSU include a positive autologous serum skin test (ASST), basophil release assay (basophil histamine release assay-BHRA or basophil activating test-BAT), and detection of specific autoantibodies. Compared to other forms of CSU, type IIb aiCSU is often associated with higher disease activity, comorbid autoimmune disorders (such as autoimmune thyroid disease), and a lower response rate to standard antihistamine therapy [5–7]. Recent advancements in CSU management, particularly with second-generation H1 antihistamines like bilastine, offer significant therapeutic benefits. Bilastine is highly effective in reducing symptoms and improving Urticaria Activity Scores (UAS) with minimal side effects, owing to its lack of blood-brain barrier penetration and hepatic or renal metabolism, making it suitable for long-term use across diverse patient populations [8,9].

Despite the effectiveness of bilastine, some patients remain inadequately controlled [10]. Thus, further research is essential to better understand the full potential of bilastine in managing CSU, particularly in patients with autoimmune-related CSU (aiCSU), as well as to address any gaps in knowledge regarding its impact on patient-reported outcomes and quality of life improvements. This study aims to measure clinical and quality of life improvement after bilastine treatment, as well as to identify prognostic factors for treatment response among patients with aiCSU in a Vietnamese hospital setting.

## Materials and methods

### Study design

This case series was conducted at the Urticaria Clinic of the Vietnam National Dermatology and Venereology Hospital from June 2023 to March 2024. The study initially included 36 patients diagnosed with aiCSU. However, 2 patients were excluded from the final analysis: one patient was lost to follow-up due to distance from the clinic, and another patient switched to a different antihistamine after expressing a preference not to continue bilastine treatment following a lack of response to the initial dose. This left a total of 34 patients for analysis. Inclusion criteria required the presence of wheals or angioedema, where wheals were defined as red or pink raised areas lasting less than 24 hours, and angioedema as swelling of subcutaneous or submucosal tissues lasting up to 72 hours. Symptoms had to occur spontaneously and persist daily or nearly daily for over six weeks. Additionally, to meet the criteria for type IIb aiCSU, patients were required to have both a positive BHRA and a positive ASST. Patients with pure chronic inducible urticaria (CIndU) or severe acute conditions were excluded from the study. Written informed consent was obtained from all participants, and the study was approved by the Institutional Review Board of Hanoi Medical University (approval number: 865/GCN-HDDDDNCYSH-DHYHN), adhering to Good Clinical Practice and local regulations.

### Procedure

Patients were treated with bilastine (Bilaxten 20 mg) tablets produced by Menarini, with dosing adjustments made based on Urticaria Control Test (UCT) scores. Patients were assessed every two weeks. For those with poor control (UCT < 12), the bilastine dose was progressively increased, up to 1–4 times the standard dose every two weeks. For patients achieving good control (UCT = 12–15), the current dose was maintained and monitored. In cases of complete control (UCT = 16), the effective dose was maintained for the entire 8-week follow-up period to ensure sustained symptom management. Patients with CSU who also had comorbid CIndU were advised on avoiding known triggers that could exacerbate CIndU symptoms, such as cold exposure or physical factors. This stepwise approach, tailored to individual patient needs, provided personalized dosing to optimize symptom control and quality of life throughout the treatment period [11]. After 8-week treatment period, patients continued to receive ongoing treatment for CSU as their treatment plan.

### Measurement

Patient data were measured at baseline and subsequently at 2, 4, 6, and 8 weeks after baseline to assess the progression of treatment responses and changes in disease activity and quality of life over time (S1 File).

Data on demographic information (age, gender), clinical characteristics, laboratory findings, and quality of life (QoL) were collected using a structured medical record. Clinical data were gathered by dermatologists trained to ensure consistency. This included allergy history, autoimmune disease history, and urticaria history (acute/chronic). The presence of CIndU was assessed through challenge tests for dermographism, cold urticaria, and cholinergic urticaria, while delayed pressure urticaria, a subtype of CIndU, was diagnosed based on clinical examination. Information on the presence of angioedema (affecting eyes, lips, limbs, throat, or none), itching severity (mild, moderate, or severe), the number of days with urticaria per week, and wheal duration was also recorded.

Laboratory data included complete blood count with differential, where eosinopenia was defined as <50 cells/mL, basopenia as <10 cells/mL. Additional tests included C-reactive protein (CRP; normal range <5 UI/mL), anti-thyroid peroxidase antibodies (IgG anti-TPO, normal range, < 5.61 UI/mL), antinuclear antibodies (ANA), and total serum IgE level (UI/mL, normal range 40–100 UI/mL). ASST followed the EAACI taskforce protocol. Patients discontinued antihistamines at least three days prior and systemic corticosteroids one month before the test. Venous blood was centrifuged, and serum was injected intradermally (0.05 ml), alongside saline as a negative control and histamine as a positive control. A positive result was defined by a serum-induced wheal ≥1.5 mm larger than the saline-induced wheal with erythema [12]. The BHRA was performed by RefLab ApS, Denmark, using donor basophils treated to remove surface IgE. Cells were incubated with patient serum, and histamine release was measured using the ortho-phthaldialdehyde method, with >16.5% histamine release considered positive [13].

To assess treatment response, the Urticaria Control Test (UCT) was used at multiple time points: after 2 weeks (UCT2), 4 weeks (UCT4), 6 weeks (UCT6), and 8 weeks (UCT8) of treatment. UCT scores were categorized as follows: 1) Poor control: UCT < 12, indicating failure with antihistamine dosing ; 2) Good control: UCT = 12 - 15; and 3) Complete control: UCT = 16. This classification allowed for a detailed evaluation of treatment efficacy, particularly in identifying cases of antihistamine treatment failure at standard doses [14].

Disease activity was assessed using the Weekly Urticaria Activity Score (UAS7), which rates the number of wheals and the intensity of itching over seven days, with a maximum score of 21 for each symptom and a total score of 42 for the entire instrument. Patients were classified into four groups based on disease severity: clear (UAS7 = 0), very mild and mild (UAS7 = 1–15), moderate (UAS7 = 16–27), and severe (UAS7 = 28–42) [4]. To evaluate treatment effectiveness, patient responses were categorized into three groups: Complete response (UAS7 = 0 and UCT = 16), Partial response (UAS7 < baseline UAS7 score and UCT = 12–15), and No response (UAS7 ≥ baseline UAS7 score or UCT < 12). Side effects of different doses were recorded including drowsiness, insomnia, dizziness and other (if any).

QoL was evaluated at baseline and after the 8-week treatment period, in accordance with the study protocol, using the Chronic Urticaria Quality of Life Questionnaire (CU-Q2oL). This 23-item tool assesses the impact of CSU on aspects such as pruritus, swelling, daily activities, sleep, limitations, and look [15]. Scores were calculated for each domain and summed to provide an overall QoL score, with higher scores indicating a greater negative impact on quality of life.

### Statistical analysis

Data were analyzed using Stata 16.0. Descriptive statistics were used to summarize the demographic, clinical, and laboratory characteristics of the patients. Continuous variables were compared between groups using the Kruskal-wallis test, while categorical variables were analyzed using the Fisher’s exact test. Repeated measures analysis was performed to evaluate changes in UAS7 scores, QoL scores, and other clinical parameters across these time points. Effect sizes were calculated using Cohen’s d to assess the magnitude of change in UAS7 and QoL scores over time. Cohen’s d values were interpreted as follows: 0.2 for small effect, 0.5 for medium effect, and 0.8 for large effect. Generalized estimating equation models were performed to measure associated factors with UAS7 and QoL score improvements. A p-value of less than 0.05 was considered statistically significant.

## Results

Table 1 included demographic and clinical characteristics of 34 CSU patients, with 70.6% being female. The mean age was 39.5 years, and the mean duration of the current urticaria episode was 29.9 weeks. A history of allergy and autoimmune disease was present in 26.5% and 8.8% of patients, respectively. Most patients had no history of urticaria (64.7%), while 23.5% had history of chronic urticaria. Comorbid CIndU was noted in 2.9% of patients. Angioedema was present in 32.3% of patients, with the lip being the most affected area (29.4%). Itching severity was classified as moderate in 44.1% and severe in 41.2%. Most patients experienced urticaria for 7 days per week (76.5%), and the duration of wheals ranged from 1 to 6 hours in 44.1%. The majority had a history of CSU treatment (64.7%).

**Table 1:** Demographic, Clinical Characteristics, Laboratory Findings, and Treatment Responses of CSU Patients.

Characteristics		N	Percentage (%)
**Gender, Female**		24	70.6
**History of allergy**		9	26.5
**History of autoimmune disease**		3	8.8
**History of urticaria**	No	22	64.7
	Acute	4	11.8
	Chronic	8	23.5
**Presence of comorbid CIndU**		1	2.9
**Presence of angioedema**		11	32.3
**Position of angioedema**	Eye	8	23.5
	Lip	10	29.4
	Limbs	5	14.7
	Throat	1	2.9
**Itching severity**	Mild	5	14.7
	Moderate	15	44.1
	Severe	14	41.2
**Number of days with urticaria per week**	< 5 days	8	23.5
	≥5 days	26	76.5
**Duration of wheal**	< 1 hour	3	8.8
	1-6 hours	15	44.1
	6-12 hours	12	35.3
	12- < 24 hours	4	11.8
**History of CSU treatment**	No	2	5.9
	Unknown	10	29.4
	Yes	22	64.7
**Eosinopenia**		7	20.6
**Basopenia**		3	8.8
**Total serum IgE level**	Low	4	11.8
	Normal	6	17.7
	High	24	70.6
**ANA positive**		5	15.2
**IgG anti-TPO positive**		2	5.9
**Highest treatment dose**	Standard dose	16	47.1
	x2 dose	9	26.5
	x3 dose	3	8.8
	x4 dose	1	2.9
	No response with x4 dose	5	14.7
		**Mean (SD)**	**Median (IQR)**
Age (years)		39.5 (15.5)	37 (17-79)
Duration from the first onset of urticaria (years)		5.8 (10.7)	0 (0-53)
Duration of current episode (weeks)		29.9 (47.8)	16 (6-260)
Eosinophil (cells/mL)		143.0 (114.3)	114.6 (18.3-498.8)
Basophil (cells/mL)		34.6 (22.4)	27.6 (6.1-100.1)
CRP (UI/mL)		2.8 (3.8)	1.4 (0.1-17.8)
Total serum IgE (UI/mL)		273.4 (253.1)	236.0 (11.7-1338)
IgG anti-TPO/IgE ratio		0.03 (0.09)	0.003 (0.00-0.04)

*Abbrev: BHRA: Basophil Histamine Release Assay; UAS7: Urticaria Activity Score; CIndU: Chronic inducible urticaria; CSU: Chronic Spontaneous Urticaria*

Eosinopenia and basopenia were present in 20.6% and 8.8% of patients, respectively. Low total serum IgE level was found in 11.8% of patients. Treatment doses varied, with 47.1% of patients achieving a complete response at the standard dose of bilastine. Dose escalation was required for some patients, and 14.7% of the patients did not achieve a response even at the highest dose of x4. Mean values for eosinophils, basophils, CRP, total serum IgE, and IgG anti-TPO are also provided.

Table 2 shows significant improvements in both disease activity and QoL among CSU patients over 8 weeks of treatment. The UAS7 score dropped from 28.9 at baseline to 2.9 after 8 weeks, showing a large effect size (ES = 2.83). Total QoL scores also improved from 44.9 at baseline to 25.3 after 8 weeks, with an effect size of 1.66. Specific QoL domains, such as pruritus, swelling, life activities, sleep problems, limits, and looks all showed significant reductions, with effect sizes ranging from 0.84 to 1.98.

**Table 2:** Disease activity and quality of life among CSU patients.

Characteristics	Baseline	After 2w			After 4w			After 6w			After 8w		
	Mean (SD)	Mean (SD)	Diffa	ESb	Mean (SD)	Diffa	ESb	Mean (SD)	Diffa	ESb	Mean (SD)	Diffa	ESb
UAS7 total	28.9 (10.5)	11.7 (12.0)	−17.1*	1.52	5.7 (11.1)	−23.1	2.13	4.3 (9.4)	−24.6	2.47	2.9 (7.6)	−25.9	2.83
CU-Q2oL Total	44.9 (16.1)	–			–			–			25.3 (4.5)	−19.6*	1.66
CU-Q2oL Pruritus	5.5 (2.1)	–			–			–			2.4 (0.7)	−3.2*	1.98
CU-Q2oL Swelling	3.2 (1.7)	–			–			–			2.0 (0.2)	−1.1*	0.99
CU-Q2oL Life activities	12.4 (5.5)	–			–			–			6.4 (1.2)	−6.1*	1.51
CU-Q2oL Sleep problems	9.3 (4.9)	–			–			–			5.5 (1.3)	−3.9*	1.06
CU-Q2oL Limits	5.9 (2.1)	–			–			–			3.2 (0.6)	−2.7*	1.75
CU-Q2oL Looks	8.6(4.1)	–			–			–			5.9 (1.9)	−2.7*	0.84

*Abbrev: UAS7: Urticaria Activity Score in 7 days; UCT: Urticaria Control Test; CU-Q*_*2*_*oL: Chronic Urticaria Quality of Life Questionnaire;* ^*a*^ *Compare to baseline score;*^*b*^Cohen’d effect size; *p < 0.05

**Fig 1:**
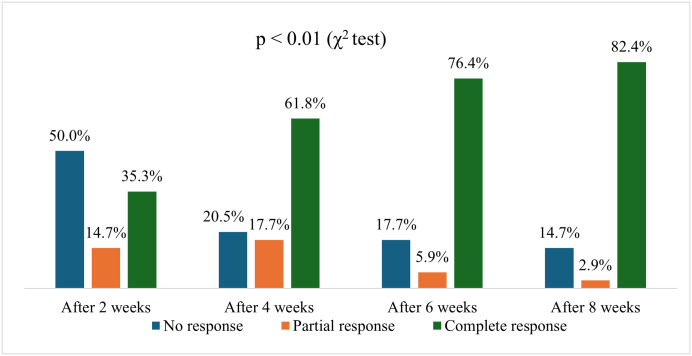
a) Treatment response; b) UAS7 categories; and c) UCT categories after 2, 4, 6 and 8 weeks (p < 0.01).

**Fig 2:**
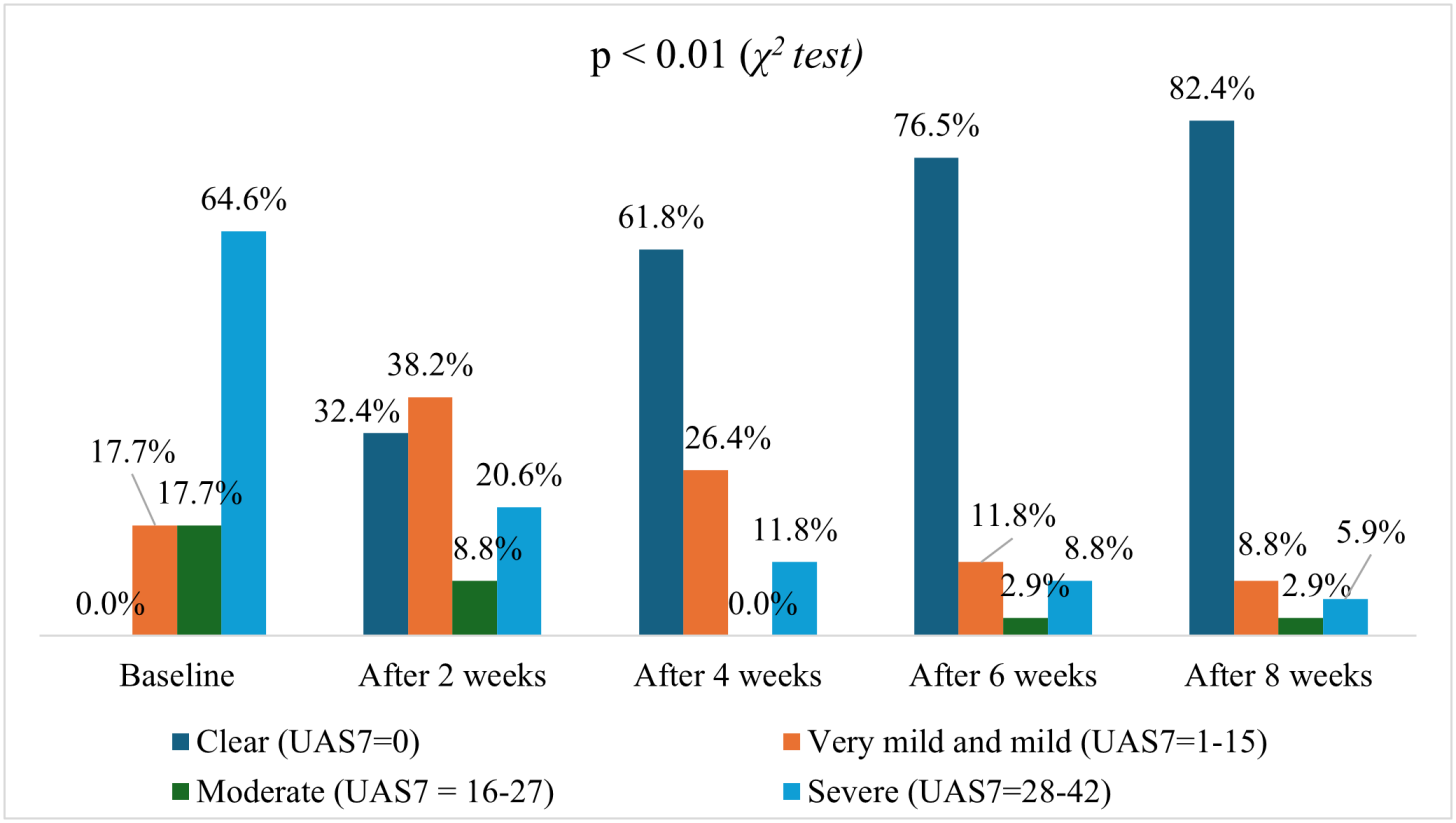
UAS7 categories after 2, 4, 6 and 8 weeks (p<0.01).

**Fig 3:**
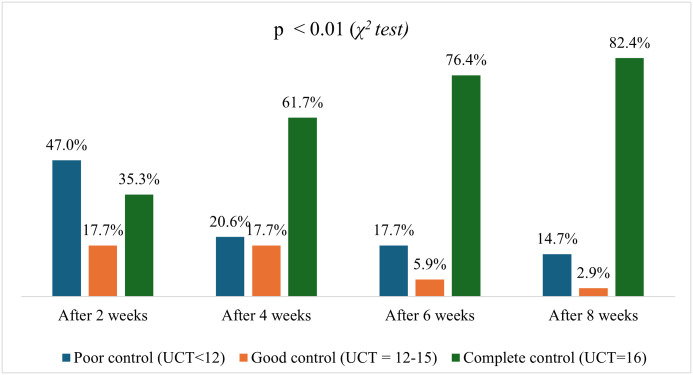
UCT categories after 2, 4, 6 and 8 weeks (p<0.01).

Fig 1 indicates that, over the course of treatment, there was a significant improvement in treatment effectiveness. Regarding UAS7 scores over 8 weeks, patients progressively shifted from severe to clear status. By week 8, 82.4% of patients were clear, while severe cases dropped from 64.6% at baseline to 5.9% (p < 0.01). (Fig 2) Similarly, regarding UCT score, at the 2-week mark, 47.0% of patients remained in the poor control category, while 17.7% achieved good control, and 35.3% reached complete control. By week 4, the proportion of patients with poor control decreased to 20.6%, those with good control slightly declined to 17.7%, and 61.7% attained complete control. Further improvements were observed at week 6, with only 17.7% in poor control, 5.9% in good control, and a significant increase to 76.4% in complete control. At the end of the 8-week period, 82.4% of patients achieved complete control, with only 14.7% remaining in poor control and 2.9% in good control. (Fig 3) The trend indicates a progressive improvement in symptom control over time, with a significant shift towards complete control by the end of the treatment period (p < 0.01).

Table 3 summarizes the side effects observed in patients during the study. Drowsiness was the most common side effect, reported by three patients at a dose escalation to x2. Insomnia was reported by one patient at the x2 dose, while dizziness was reported by one patient at the x3 dose. All side effects were mild and transient, not affecting the continuation of treatment.

**Table 3:** Side effect.

Side effect	Dose	Number of cases
Drowsiness	x2	3
Insomnia	x2	1
Dizziness	x3	1

The multivariable analysis in Table 4 identified several clinical and laboratory factors associated with urticaria activity (UAS7 score), disease control (UCT score), and treatment response over time. Patients with autoimmune disease had significantly higher UAS7 scores (β = 9.34; 95% CI: 3.44 to 15.24), and those with basopenia also showed increased disease activity (β = 11.93; 95% CI: 7.04 to 16.82). In contrast, having symptoms ≥5 days per week was associated with a significant reduction in UAS7 score (β = –5.54; 95% CI: –9.85 to –1.24). Baseline UAS7 score was positively associated with higher follow-up UAS7 (β = 0.49; 95% CI: 0.35 to 0.62), indicating persistent disease activity.

**Table 4:** Factors associated with UAS7 score, good disease control according to UCT score and complete response over time.

Characteristics	UAS7 score	Partial/poor disease control according to UCT score	No/Partial Response
	Coef. (95% CI)	OR (95% CI)	OR (95% CI)
Age (years)	−0.11 (–0.23, 0.01)	0.98 (0.92, 1.04)	1.00 (0.95, 1.06)
Male (vs Female^a^)	−2.99 (–6.42, 0.44)	0.72 (0.17, 3.12)	1.64 (0.51, 5.21)
History of allergy (vs No^a^)	−0.94 (–4.26, 2.39)	2.41 (0.59, 9.80)	1.94 (0.64, 5.88)
Acute urticaria (vs None^a^)	0.57 (–4.35, 5.49)	8.72 (0.85, 89.76)	7.37 (1.08, 50.56)*
Chronic urticaria (vs None^a^)	0.85 (–3.48, 5.17)	5.92 (0.67, 52.60)	3.13 (0.62, 15.78)
Autoimmune disease (vs No^a^)	9.34 (3.44, 15.24)*	28.16 (2.79, 284.09)*	10.66 (1.57, 72.54)*
Disease duration (years)	−0.01 (–0.22, 0.19)	0.91 (0.83, 1.00)	0.95 (0.88, 1.01)
Having symptoms ≥5 days/week (vs < 5 days/week^a^)	–5.54 (–9.85, –1.24)*	0.06 (0.007, 0.45)*	0.05 (0.006, 0.32)*
Eosinopenia (vs No^a^)	−2.01 (–5.62, 1.59)	0.47 (0.07, 3.31)	0.72 (0.15, 3.41)
Basopenia (vs No^a^)	11.93 (7.04, 16.82)*	25.72 (3.44, 192.24)*	17.19 (3.00, 98.43)*
IgE normal (vs IgE low^a^)	−2.91 (–8.50, 2.69)	0.12 (0.01, 1.10)	0.08 (0.01, 0.57)*
IgE high (vs IgE low^a^)	−4.19 (–9.58, 1.20)	0.03 (0.001, 0.44)*	0.04 (0.004, 0.41)*
Baseline UAS7 score	0.49 (0.35, 0.62)*	1.19 (1.08, 1.30)*	1.14 (1.06, 1.23)*

*Statistically significant at p < 0.05; Abbreviations: UAS7 – Urticaria Activity Score over 7 days; UCT – Urticaria Control Test; OR – Odds Ratio; CI – Confidence Interval; ^*a*^ *reference group.*

Regarding disease control, autoimmune disease (OR = 28.16; 95% CI: 2.79 to 284.09), basopenia (OR = 25.72; 95% CI: 3.44 to 192.24), and high baseline UAS7 score (OR = 1.19; 95% CI: 1.08 to 1.30) were associated with poor UCT control. Similarly, autoimmune disease (OR = 10.66; 95% CI: 1.57 to 72.54), acute urticaria history (OR = 7.37; 95% CI: 1.08 to 50.56), basopenia (OR = 17.19; 95% CI: 3.00 to 98.43), and higher baseline UAS7 (OR = 1.14; 95% CI: 1.06 to 1.23) were associated with no or partial treatment response. Conversely, patients with high IgE levels had lower odds of poor disease control (OR = 0.03; 95% CI: 0.001 to 0.44) and incomplete response (OR = 0.04; 95% CI: 0.004 to 0.41).

## Discussion

This study evaluated the clinical effectiveness of bilastine treatment and its impact on the QoL in patients with type IIb aiCSU over an 8-week period. The findings demonstrate significant improvements in disease activity, control, and QoL among the studied population. The majority of patients showed marked clinical improvement, with a substantial reduction in UAS7 scores, a higher rate of good disease control based on UCT scores, and enhanced QoL.

The response rate to bilastine in this study was high, with 82.4% of patients achieving complete response by week 8; however, only 47% responded to the standard dose, while 52.9% remained refractory, highlighting the treatment challenges in type IIb aiCSU. Even with dose escalation to x4, 14.7% of patients did not achieve full control, underscoring the resistant nature of aiCSU. This contrasts with a study in India, where 73.48% of CSU patients responded to the standard dose [16], likely reflecting differences in disease mechanisms, as aiCSU has been shown to have poor response to standard-dose H1 antihistamines [17]. Although histamine is the primary mediator in urticaria and H1 antihistamines are the foundational treatment, meta-analyses indicate that only about 38.6% of CSU patients respond to standard doses [18]. Markers associated with type IIb aiCSU-such as high disease activity, positive BHRA/BAT, low eosinophil/basophil counts, low IgE, and elevated IgG anti-TPO levels-are linked to poor antihistamine response [19,20]. Prior research showed that type IIb aiCSU, marked by autoimmune activity, often responds poorly to antihistamines but shows improved outcomes with immunosuppressive agents like cyclosporine [21].

The significant reduction in UAS7 scores in our study, from a baseline mean of 28.9 to 2.9 by week 8, aligns with previous research demonstrating bilastine’s efficacy in managing CSU. Similar findings were reported by Chakraborty et al. (2023), where refractory CSU patients saw UAS7 scores drop from 25.47 to 6.47 after 12 weeks of bilastine treatment [22]. Comparative studies further support bilastine’s superior efficacy over other antihistamines; for example, bilastine showed greater UAS7 reductions than levocetirizine from day 14 and achieved better outcomes than fexofenadine with up-dosing [16,23]. The non-sedative nature and unique receptor interactions of bilastine likely contribute to its effectiveness and patient compliance, resulting in sustained improvements in disease activity [9,23]. Similarly, UCT scores showed significant improvement over the 8-week treatment, with poor disease control (UCT < 12) dropping from 47.1% at baseline to only 14.7% by week 8, and 85.3% of patients achieving good control (UCT ≥ 12). This aligns with studies like those from India, where bilastine raised UCT scores from 9 to 13.05 over four weeks, with 73.48% classified as responders [16,24]. Bilastine’s effectiveness over other antihistamines in UCT improvement, such as its superior outcomes to levocetirizine and better tolerance compared to fexofenadine, has been documented [23,25]. Our study supports these findings, as dose escalation of bilastine further enhanced symptom control in non-responders, without additional sedation, consistent with the efficacy seen in previous research [8,26].

QoL was remarkably improved in patients with CSU treated with bilastine, as measured by the CU-Q2oL. The total CU-Q2oL score decreased from 44.9 at baseline to 25.3 by week 8, reflecting a marked improvement in overall well-being. This is consistent with findings from another study, which demonstrated that bilastine significantly improved QoL compared to fexofenadine, with a CU-Q2oL score of 32.38 ± 5.83 for bilastine versus 38.71 ± 5.92 for fexofenadine (p < 0.05) [27]. This suggests that bilastine is more effective than some other antihistamines in improving QoL for CSU patients. Moreover, a systematic review and meta-analysis of bilastine’s effects on chronic urticaria confirmed its efficacy in enhancing Dermatology Life Quality Index (DLQI) scores, which are closely related to CU-Q2oL. The meta-analysis found significant improvements in QoL indices, further supporting bilastine’s role in improving patient well-being [28]. In our study, specific QoL domains, including pruritus, swelling, life activities, limits and sleep, all showed significant improvements, with pruritus and life activities demonstrating the greatest reduction in impact. This highlights bilastine’s effectiveness not only in managing the physical symptoms of CSU but also in alleviating the psychosocial burden of the disease.

In our study, several factors, including basopenia, low total serum IgE levels, high baseline UAS7 score and a history of autoimmune disease, were found to be significantly associated with treatment response in CSU patients treated with bilastine. Basopenia, defined by low basophil counts in peripheral blood, is commonly observed in CSU patients and is believed to result from the migration of basophils to the skin as part of the inflammatory process. This migration reflects the disease’s underlying pathology, where basophils contribute to the skin’s inflammatory environment. Studies have shown that restoration of peripheral basophil counts is associated with symptom improvement in CSU patients treated with antihistamines, including bilastine [29]. Patients with basopenia were more likely to achieve partial/poor disease control and more likely to show no or partial response to treatment. This aligns with previous research suggesting that basopenia may serve as a marker for more severe or refractory CSU, where the inflammation is more intense, and the migration of basophils is more pronounced [30,31]. Improvement in basophil counts after treatment could potentially serve as an indicator of treatment efficacy.

Normal or elevated total serum IgE levels were found to be another factor associated with treatment response in our cohort. Specifically, our study showed that higher IgE levels were linked to better treatment outcomes among patients aiCSU treated with bilastine. Previous studies have highlighted the role of IgE levels in predicting treatment response, particularly for therapies such as omalizumab, an anti-IgE monoclonal antibody [20,32]. Patients with low IgE levels—especially those with type IIb aiCSU, tend to respond poorly to omalizumab [33–35]. Although the direct relationship between IgE levels and bilastine response has not been extensively investigated, the distinct pathophysiology in low-IgE aiCSU patients may influence their responsiveness to antihistamines like bilastine.

A history of autoimmune disease significantly impacted treatment response to bilastine in our CSU patient cohort. Specifically, among the patients with autoimmune disease, comorbidities included autoimmune thyroid disease (2 cases), rheumatoid arthritis (2 cases), and vitiligo (1 case). Autoimmune thyroid disease, frequently found to coexist with CSU, does not yet have an established causal link to CSU, although there is some evidence suggesting that thyroid hormone supplementation might benefit CSU management [36]. The presence of autoimmune conditions appears to correlate with a poorer response to standard antihistamine treatments like bilastine in CSU patients, possibly due to the complex, underlying autoimmune mechanisms characteristic of aiCSU. This aligns with findings from previous studies, which indicate that markers of autoimmunity, such as a positive BHRA + , are associated with reduced responsiveness to antihistamines but improved outcomes with immunosuppressive agents, such as cyclosporine [37]. In our study, patients with autoimmune comorbidities had a higher disease burden, with higher UAS7 scores and lower rates of achieving good disease control compared to those without autoimmune conditions. These findings underscore the potential need for more targeted treatments in aiCSU patients, as their response to antihistamines may be limited. The identification of predictors like basopenia and low IgE levels could guide clinicians in personalizing treatment, helping to identify patients who may require alternative approaches, such as immunosuppressants, to achieve optimal control of their symptoms.

The findings from this study have important clinical implications. Bilastine was shown to be highly effective in managing CSU, with most patients achieving significant improvements in disease activity and QoL within 8 weeks. The identification of factors such as basopenia and low IgE levels as predictors of treatment response may help clinicians personalize treatment strategies and better identify patients who may require more aggressive or alternative therapies.

This study has several limitations. First, it was conducted as a single-center case series with a relatively small sample size, which may limit the generalizability of the findings. Second, the follow-up period was limited to 8 weeks, which may not capture the long-term effectiveness and safety of bilastine. Additionally, the study did not include a placebo control group, which makes it difficult to fully attribute the observed improvements to bilastine alone. Furthermore, our definition aiCSU relied on two out of three criteria (positive BHRA and ASST), which may not fully capture all cases of aiCSU. Future studies should consider a larger, multicenter cohort with a longer follow-up period to confirm these findings and explore the long-term benefits of bilastine in aiCSU management.

## Conclusion

In conclusion, bilastine demonstrated substantial efficacy in reducing disease activity, improving symptom control, and enhancing QoL in patients with type IIb aiCSU. While the results support the use of bilastine as an effective therapeutic option, approximately 50% of patients did not respond to the standard dose, and 14.7% were unable to achieve full control even at the maximum dose of x4. Future research is warranted to explore long-term benefits and optimize treatment strategies, particularly for aiCSU patients who remain refractory to antihistamine therapy.

## Supporting information

S1 File Questionnaire. (DOCX)
