# Supplementary material for: Clinical and quality of life improvement after bilastine treatment among patients with autoimmune chronic spontaneous urticaria
Source: PLoS One. 2025 Aug 25;20(8):e0326445. doi: 10.1371/journal.pone.0326445 (PMC12377582; doi:10.1371/journal.pone.0326445)
Supplement: S1 File — (DOCX) [file pone.0326445.s001.docx]

**CLINICAL, LABORATORY CHARACTERISTICS AND TREATMENT RESPONSE OF AUTOIMMUNE CHRONIC URTICARIA**

**1. GENERAL INFORMATION**

1.1. Research Patient ID: ………………
1.2. Full Name: …………………………………………… Phone Number: …………….…
1.3. Gender: ☐ Male ☐ Female
1.4. Year of Birth: ………………….
1.5. Patient ID (HIS): ………………….
1.6. Chronic Urticaria Management ID: ………………….
1.7. Date of Examination: ………………….
1.8. Address: ☐ Hanoi ☐ Other Province: ……………..

**2. MEDICAL HISTORY**

**Personal History:**
2.1. History of Urticaria:

- Previous history of urticaria:
  ☐ No ☐ Acute Urticaria ☐ Chronic Urticaria
- Number of years since the first onset of urticaria symptoms: ………… (years)

2.2. History of associated allergic diseases:
☐ No ☐ Specific disease: …………

2.3. History of autoimmune diseases:
☐ No ☐ Specific disease: …………

**Family History:**
2.4. Family history of chronic urticaria:
☐ No ☐ Specific disease: …………

2.5. Family history of allergic diseases:
☐ No ☐ Specific disease: …………

2.6. Family history of autoimmune diseases:
☐ No ☐ Specific disease: …………

**3. CLINICAL CHARACTERISTICS**

3.1. Duration of the current urticaria episode: …………………. (weeks)

3.2. Presence of inducible chronic urticaria:
☐ No ☐ Specific type: …………

3.3. Associated angioedema:
☐ No ☐ Eyes ☐ Lips ☐ Hands and Feet ☐ Other: …………

3.4. Number of days with urticaria symptoms per week: …………

3.5. Itching severity:
☐ No itching ☐ Mild itching ☐ Moderate itching ☐ Severe itching

3.6. Duration of wheal persistence:
☐ <1 hour ☐ 1-6 hours ☐ 6-12 hours ☐ 12 to <24 hours

**4. LABORATORY CHARACTERISTICS**

| **No.** | **Test Name** | **Unit** | **Result** |
| --- | --- | --- | --- |
| 4.1 | Total White Blood Cell Count | Cells/L | ………… |
| 4.2 | Basophils | % | ………… |
| 4.3 | Eosinophils | % | ………… |
| 4.4 | CRP | mg/L | ………… |
| 4.5 | FT3 | UI/ml | ………… |
| 4.6 | FT4 | UI/ml | ………… |
| 4.7 | TSH | UI/ml | ………… |
| 4.8 | SGOT | UI/ml | ………… |
| 4.9 | SGPT | UI/ml | ………… |
| 4.10 | Total Serum IgE | UI/ml | ………… |
| 4.11 | IgG-anti TPO | UI/ml | ………… |
| 4.12 | ANA | - | ………… |
| 4.13 | ASST | - | ………… |
| 4.14 | BHRA | - | ………… |

**5. TREATMENT**

**Bilastine Treatment**

| **Treatment Period** | **T0** | **T2** | **T4** | **T6** | **T8** |
| --- | --- | --- | --- | --- | --- |
| Date |  |  |  |  |  |
| Bilastine Dose (taken for 2 weeks before follow-up) |  |  |  |  |  |
| UAS7 |  |  |  |  |  |
| UCT |  |  |  |  |  |
| CU-Q2oL |  |  |  |  |  |

- **Treatment response to the highest bilastine dose:**
  ☐ 20mg/day ☐ 40mg/day ☐ 60mg/day ☐ 80mg/day
  ☐ No response at 80mg/day
- **Side Effects:**
  ☐ No side effects

| **Symptoms** | **Dose at which side effect occurred** | **Severity** | **Time from onset to resolution** |
| --- | --- | --- | --- |
| Drowsiness |  |  |  |
| Headache |  |  |  |
| Dizziness |  |  |  |
| Fatigue |  |  |  |
| Other ……… |  |  |  |

**Urticaria Activity Score (UAS7)**

| **Score** | **Wheals** | **Itching** |
| --- | --- | --- |
| **0** | None | None |
| **1** | Mild (<20 wheals/24h) | Mild (not bothersome) |
| **2** | Moderate (20-50 wheals/24h) | Moderate (bothersome but does not affect daily activities or sleep) |
| **3** | Severe (>50 wheals/24h or large affected area) | Severe (intense itching affecting daily activities or sleep) |

| **Day** | **1** | **2** | **3** | **4** | **5** | **6** | **7** | **Total** |
| --- | --- | --- | --- | --- | --- | --- | --- | --- |
| **T0** |  |  |  |  |  |  |  |  |
| **T2** |  |  |  |  |  |  |  |  |
| **T4** |  |  |  |  |  |  |  |  |
| **T6** |  |  |  |  |  |  |  |  |
| **T8** |  |  |  |  |  |  |  |  |

**Urticaria Control Test (UCT) – Assessment of Disease Control in the Past 2 Weeks**

| **Question** | **Very much** | **A lot** | **Moderate** | **Little** | **None** |
| --- | --- | --- | --- | --- | --- |
| How much have you suffered from urticaria symptoms (itching, wheals, swelling) in the past 2 weeks? | 0 | 1 | 2 | 3 | 4 |
| How much has urticaria affected your quality of life in the past 2 weeks? | 0 | 1 | 2 | 3 | 4 |
| How often has your treatment been insufficient to control urticaria in the past 2 weeks? | 0 | 1 | 2 | 3 | 4 |
| How well has your urticaria been controlled in the past 2 weeks? | 0 | 1 | 2 | 3 | 4 |

|  | **T2** | **T4** | **T6** | **T8** |
| --- | --- | --- | --- | --- |
| **UCT Score** |  |  |  |  |
|  |  |  |  |  |

**Chronic Urticaria Quality of Life Questionnaire (CU-Q2oL)**

**In the past 14 days, how much have you been bothered by the following symptoms?**

| **Symptoms** | **None** | **Little** | **Moderate** | **A lot** | **Very much** |
| --- | --- | --- | --- | --- | --- |
| 1. Itching | 1 | 2 | 3 | 4 | 5 |
| 2. Swelling | 1 | 2 | 3 | 4 | 5 |
| 3. Eye swelling | 1 | 2 | 3 | 4 | 5 |
| 4. Lip swelling | 1 | 2 | 3 | 4 | 5 |

**In the past 14 days, how frequently has urticaria affected the following daily activities?**

| **Activity** | **None** | **Rarely** | **Occasionally** | **Frequently** | **Very frequently** |
| --- | --- | --- | --- | --- | --- |
| Work | 1 | 2 | 3 | 4 | 5 |
| Physical activities | 1 | 2 | 3 | 4 | 5 |
| Sleep | 1 | 2 | 3 | 4 | 5 |
| Leisure time | 1 | 2 | 3 | 4 | 5 |
| Social activities | 1 | 2 | 3 | 4 | 5 |
| Eating | 1 | 2 | 3 | 4 | 5 |

**In the past 14 days, how often have you experienced difficulties due to urticaria?**

| **Issue** | **None** | **Rarely** | **Occasionally** | **Frequently** | **Very frequently** |
| --- | --- | --- | --- | --- | --- |
| Difficulty falling asleep | 1 | 2 | 3 | 4 | 5 |
| Waking up at midnight | 1 | 2 | 3 | 4 | 5 |
| Daytime fatigue due to lack of sleep | 1 | 2 | 3 | 4 | 5 |
| Difficulty concentrating | 1 | 2 | 3 | 4 | 5 |
| Stress | 1 | 2 | 3 | 4 | 5 |
| Distress | 1 | 2 | 3 | 4 | 5 |
| Limited food choices | 1 | 2 | 3 | 4 | 5 |
| Annoyance due to urticaria symptoms | 1 | 2 | 3 | 4 | 5 |
| Embarrassment in public places | 1 | 2 | 3 | 4 | 5 |
| Need to use cosmetics to cover symptoms | 1 | 2 | 3 | 4 | 5 |
| Limited clothing choices | 1 | 2 | 3 | 4 | 5 |
| Limited participation in sports | 1 | 2 | 3 | 4 | 5 |
| Enduring side effects of treatment | 1 | 2 | 3 | 4 | 5 |

|  | **T0** | **T8** |
| --- | --- | --- |
| **CU-Q2oL Score** |  |  |
|  |  |  |
